# Hemophagocytic Syndrome Associated with Hodgkin's Lymphoma First Presenting as Fever and Pancytopenia

**DOI:** 10.1155/2010/759651

**Published:** 2010-11-07

**Authors:** Ramon Andrade Bezerra de Mello, Elsa Fonseca, Manuela Brochado, João Manuel Quinaz

**Affiliations:** ^1^Internal Medicine Department, São João Hospital, 4200-319 Porto, Portugal; ^2^Department of Medicine, Faculty of Medicine, Porto University, 4200-319 Porto, Portugal; ^3^Surgical Pathological Department, São João Hospital, 4200-319 Porto, Portugal; ^4^Department of Surgical Pathology, Faculty of Medicine, Porto University, 4099-002 Porto, Portugal; ^5^Clinical Hematology Department, São João Hospital, 4200-319 Porto, Portugal

## Abstract

*Background*. Hemophagocytic syndrome (HPS) is characterized by a hyperinflammatory reaction followed by alteration in cytotoxic function of Th1 lymphocytes and natural killer cells. We report a rare case of a patient that presented with fever and pancytopenia due to HPS associated with Hodgkin's lymphoma (HL). *Case Report*. A 69-year-old Caucasian woman was admitted presenting with complaints of fever, seizures, and low back pain that had lasted for two weeks. Laboratorial data showed pancytopenia. Bone marrow biopsy revealed infiltration by Reed-Sternberg cells and hemophagocytosis signs. Imaging studies showed mediastinal lymph nodes (stage IV B). She had been treated with ABVD (doxorubicin, bleomycin, vinblastine, and dacarbazine) followed by a good response. *Conclusion*. HPS associated with HL is a very rare and lethal disease, with mortality rates of about 15% to 60%. The prompt diagnosis of the underlying lymphoma may be an important strategy for optimizing the clinical approach and outcome.

## 1. Introduction

Hemophagocytic syndrome (HPS) is characterized by the proliferation of benign macrophages responsible for extensive phagocytosis of hematopoietic cells due to a hyperinflammatory reaction that may cause alteration in cytotoxic function of T lymphocytes and natural killer cells [1, 2]. One of the most consistent findings is low or absent cytotoxicity activity of natural killer (NK) cells. This biological event is related to a defective production of cytokines and anomalous inflammatory reaction that change the normal behavior of the immune system leading to deregulated cell signaling. Some studies suggest that the lack of perforins, a hydrophobic protein that is packaged into specialized cytotoxicity granules of human natural killer cells (CD56^low^, CD8^−^), natural killer T cells (CD56^low^, CD8^+low^), and activated CD8^+^ T helper 1 lymphocytes (Th1), results in insufficiency to regulate or terminate a immune response [1, 3–5]. These perforins seem to act with granzymes, a serine protease, leading to induced apoptosis in the target cells. Mutation in the perforin gene accounts for about 20%–40% of hemophagocytic lymphohistiocytosis subjects [3, 6]. HPS is a clinical pathological disease described by histiocytic proliferation, fever, hepatosplenomegaly, cytopenia, altered liver function, hyperferritinemia, hypertriglyceridemia, low plasma fibrinogen levels, and/or frequently coagulopathy disorders [1, 3, 4, 7]. It can be genetic, when associated with inherited alteration of T lymphocytes and NK cells behavior, or acquired, associated with infection, mainly Epstein-Barr virus (EBV), autoimmune, or immunodeficiency status. It is rarely associated with B cell lymphoma [1, 4]. The treatment is very difficult, and some studies [3, 4, 8] report a bad therapeutic prognosis sometimes, with a clinical response to chemotherapy rate of about 65% [4]. In this paper, we report a rare case of a patient that first presented with fever and pancytopenia due to hemophagocytosis associated with Hodgkin's lymphoma (HL) and highlight the difficulties regarding diagnosis and clinical approach.

## 2. Case Report

**Figure 1:**
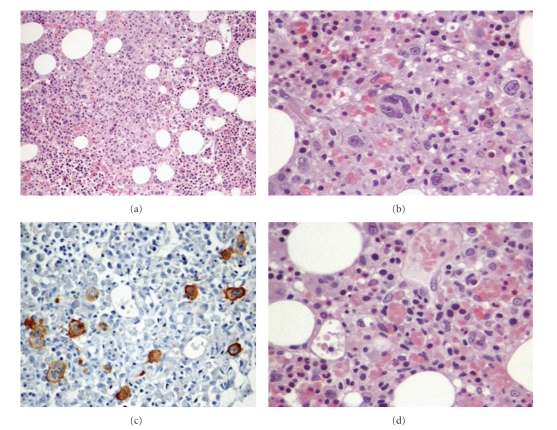
Bone marrow biopsies revealing Hodgkin's disease and hemophagocytosis. (a) Hematoxylin and eosin (HE ×100), showing the general aspect of bone marrow. (b) HE ×600, showing the Reed-Sternberg cells. (c) Blade positive to CD30. (d) HE ×600, showing in the center of figure the hemophagocytosis signs: the macrophage with erythrocytes inside.

A 69-year-old Caucasian woman was admitted to the internal medicine ward presenting with symptoms of fever, seizures, and low back pain that had lasted for two weeks, followed by asthenia and dyspnea on moderate exertion. She had a medical history of probable inflammatory oculomotor palsy (under chronic corticotherapy), systemic arterial hypertension, dyslipidemia, obesity, and hyperhomocysteinemia. She had irrelevant familial history, without consanguineous parents. On physical examination, she had good performance status, left ptosis, left oculomotor palsy, no palpable lymph node, normal cardiopulmonary and abdominal examinations, and no peripheral edemas. The subsequent laboratorial study revealed pancytopenia, hyperglycemia, hypertriglyceridemia, hyperferritinemia, low fibrinogen levels, and high *β*-2 microglobulin levels (Table 1). The thoracic-abdominal computed tomography (CT) showed mediastinal lymph nodes swelling between 12 and 15.5 mm and mild hepatosplenomegaly. The bone marrow biopsies revealed marrow infiltration by Hodgkin's lymphoma with Reed-Sternberg cells and hemophagocytosis signs (Figure 1) (stage IV B by Ann Arbor). The patient was submitted to high-dose corticotherapy followed by chemotherapy (ABVD—doxorubicin, bleomycin, vinblastine, and dacarbazine). She had an allergic reaction to bleomycin, which was changed to cyclophosphamide. On discharge the patient retained good clinical status, without fever and other complains.

## 3. Discussion

Hodgkin's disease was first macroscopically described by Thomas Hodgkin in 1832 as a primary nodal lymphoproliferative disorder. In 1898 and 1902, Carl Sternberg and Dorothea Reed, respectively, reported the characteristic binucleated and multinucleated dysplastic giant cells located in an abundant cellular background that came to be called Reed-Sternberg cells. After 1990s, immunophenotyping and clinical data showed that this pathology basically had two distinct entities: nodular lymphocyte-predominant Hodgkin's lymphoma and classic Hodgkin's lymphoma [9]. The hemophagocytic syndrome is a very rare and lethal disease. It was first described by Scott and Robb-Smith in 1932 who named it “histiocytic medullary reticulosis”. In 1979, Riscall et al. first characterized virus-associated HPS in a case series of 19 patients. Due to the difficulty to express a correct diagnosis, the incidence of HPS is difficult to estimate; a Japanese study group has reported an annual incidence of about 1 in 800.000 [1, 10]. The mortality rate is about 15% to 60%. HPS may be associated with EBV virus in 20%–80% of cases, and it is predominant in males. The EBV infection is consistently associated with HL, and older patients with this situation have a poor prognosis and, in general, tolerate current treatments less well than young patients [11]. Some papers hypothesize that the latent membrane protein (LPM-1) released by tumor cells infected by EBV may induce the production of large amounts of Th1 cytokines by Reed-Sternberg cells, leading to hemophagocytosis [1, 6]. These mechanisms could be responsible for intracellular signaling that regulates the apoptosis and macrophage recruitment resulting in cell destruction, but it is still not clear [6]. Bhagwati et al. reported that the clinical manifestation of HPS is a result of a variable elevation of various inflammatory cytokines including interferon-*γ* (IFN-*γ*), tumor necrosis factor-*α* (TNF-*α*), interleukin-1*β* (IL-1*β*), soluble interleukine 2 receptor, and linterleukin-6 (IL-6) leading to a systemic inflammatory response syndrome [12]. In 2005 Mazodier et al. reported that low levels of interleukin 18 may be associated with secondary HPS in patients with underlying disease, because it is a strong inducer of Th-1 response, IFN-*γ* production, and stimulation of macrophages and NK cells [13]. In 2010, Aldinucci et al. reported that the nuclear factor-*κ* *β* (NF-*κ* *β*) and an altered JAK-STAT signaling pathway are part of the biological mechanisms associated with the inflammatory response of Hodgkin's disease, indirectly helping to regulate the cell death. The overexpression of the members of the TNF receptor family, like CD30 and CD40, is a hallmark of Hodgkin and Reed-Sternberg cells [14]. To confirm diagnosis of acquired HPS, five out of eight criteria should exist: fever, splenomegaly, cytopenia in more than two cell lines (hemoglobin <90 g/L or neutrophils <1 × 10^9^/L, e.g.), hypertriglyceridemia (fasting triglycerides more than 3 mmol/L) and/or hypofibrinogenemia (<1.5 g/L), ferritin more than 500 ug/L, soluble CD25 ≥ 2400 U/mL, decreased or absent NK cell activity, and hemophagocytosis in bone marrow or lymph nodes. In this case the patient had almost all of these criteria including the bone marrow alteration [15]. The main point of HPS treatment is to suppress the hyperinflammation that is responsible for threatening the patient's life and treat the principal cause of this stimulus. The corticosteroids are of fundamental importance in reducing cytokine release and inhibiting the inflammatory reaction. In 2010, Herbst et al. reported that the combination of radiotherapy and chemotherapy with ABVD may improve the tumor control and 5-year overall survival in patients with early stage Hodgkin's lymphoma, which was not the case with our patient. However, it is important to note that cardiac toxicity is associated with mediastinal radiation and with cumulative dose of chemotherapy, in particular doxorubicin [8]. The optimal treatment for patients with Hodgkin's disease and HPS has not yet been established. ABVD chemotherapy seems a reasonable choice. Patients with poorly responsive disease may be suitable for allogeneic hematopoietic stem cell transplantation with reduced intensity conditioning in order to avoid transplant-related complications and prevent sequential relapse [16].

## 4. Conclusion

Acquired hemophagocytic syndrome associated with Hodgkin's lymphoma is a rare, fatal, and sometimes underdiagnosed disease, frequently triggered by viral infections or in association with inflammatory disease [10, 17]. Thus, the prompt diagnosis of the underlying lymphoma is the most important strategy for optimizing the clinical approach and improving the outcome. Therefore, it is important that physician should be alert to and suspect this pathology in cases of patients with unexplained fever, organomegaly, and blood count alteration, such as cytopenia with more than two lines, even if there are no palpable lymph nodes.

## Figures and Tables

**Table 1:** Laboratorial data on admission to internal medicine ward: blood analysis.

Laboratorial data	Values	Reference values
Hemoglobin	7.6 g/dL	12–16
Hematocrit	23.8%	37–49
Leucocyte count	1.17 × 10^9^/L	4.0–11.0
Neutrophils	65%	53.8–69.8
Lymphocyte	16.2%	25.3–47.3
Platelets	49 × 10^9^/L	180–500
Albumin	33.5 g/L	38–51
Total protein	53.8 g/L	64–83
AST (aspartate aminotransferase)	45 U/L	10–31
ALT (alanine aminotransferase)	105 U/L	10–31
Gama glutamyltransferase	719 U/L	7–32
Alkaline phosphatase	274 U/L	38–145
Total Bilirrubin	24.6 mg/L	<12
LDH (lactate dehydrogenase) seric	417 U/L	135–225
Prothrombin time	12.8 Seconds	10.5–13.5
Activated partial thromboplastin time	28.3 Seconds	24.5–36.5
Fibrinogen level	178 mg/dL	190–400
Glucose	250 mg/dL	75–115
Urea	59 mg/dL	10–50
Creatinine	0.91 mg/dL	0.6–1.0
Ferritin	9606.00 ng/mL	14.0–233
Triglycerides	6.36 g/L	<1.50
C-reactive protein	5.5 mg/L	<3.0
*β*-2 microglobulin	6620 g/L	700–3400
